# Use of Equine-Assisted Services to Improve Outcomes Among At-Risk and Indigenous Youth: A Scoping Review

**DOI:** 10.3389/fpubh.2022.730644

**Published:** 2022-03-28

**Authors:** Laurie Haig, Kelly Skinner

**Affiliations:** School of Public Health Sciences, University of Waterloo, Waterloo, ON, Canada

**Keywords:** youth, Indigenous health, scoping review, equine-assisted services (EAS), at-risk youth

## Abstract

Equine-assisted services (EAS) are gaining popularity as ways to promote psychological health and social well-being. EAS may show particular promise as culturally appropriate initiatives for at-risk Indigenous youth, as they are thought to align well with Indigenous ways of knowing which place emphasis on relationships between the land and all living beings. We seek to better understand previous uses of EAS as initiatives for at-risk youth populations, including Indigenous populations, and learn about which outcomes have been addressed in the literature with an EAS initiative by conducting a scoping review. The review focused on initiatives targeting at-risk youth aged 10-18 years of age in Canada, Australia, New Zealand, and the United States. A total of 27 studies were included in the final analysis from all target countries except New Zealand. The target populations were further divided into four subgroups: at-risk youth, youth with mental health disorders and/or learning disabilities, youth survivors of trauma/abuse, and at-risk Indigenous youth. Overall findings of the review suggest EAS are promising approaches for achieving therapeutic and learning goals with the potential to be successful with both Indigenous youth and at-risk youth more broadly.

## Introduction

Horses have been involved in a range of medical and therapeutic interventions including among others, hippotherapy for musculoskeletal disorders (1), cerebral palsy (2, 3), Down's syndrome (4), or autism spectrum disorder (5–9), therapeutic riding for cancer recovery (10, 11) and even initiatives involving equines for weight management (12, 13). White-Lewis identified 26 therapeutic medical uses of horses (14), but research conducted as a part of this review suggests there are likely more. In a recently published consensus document Wood et al. identified 12 distinct types of equine services, covering the use of horses in therapeutic settings, learning environments and horsemanship activities (15). Their consensus document recommended the adoption of the term equine-assisted services (EAS), as the most appropriate unifying term to refer to various services where professionals use equines to help clients (15). Therefore, this paper will use the term EAS to refer in general to the various types of equine programs, or a similar term to relate to a more specific type of EAS when relevant. EAL (equine-assisted learning) will be used when referencing the specific initiative of interest in this review. Please see section EAL Definition for further details on the nature of initiatives included in this review and see Table 1 for working definitions of various equine initiatives that will be referenced in this review. It has been suggested that EAS can increase confidence, as clients learn to respect the power of the horses during their interactions. Previous examples of EAS have been found to increase client retention in addictions programs and serve as a precursor to engagement in verbal therapy (16). Clients report that the relationship with horses facilitates trust and communication, and clients are able to overcome fear, as mastery is gained over the horse. The intention of this review was to assess the potential for using horses in therapeutic initiatives with Indigenous populations, more specifically the use of equine-assisted learning (EAL) and related initiatives as therapeutic activities for Indigenous youth. Previous research has suggested a potential for EAS to be adapted in ways that are grounded in Indigenous ways of knowing which would be suitable for use with Indigenous communities. Notably, Snowshoe and Starblanket provide a framework to explain the potential value of using the Lac La Croix Indigenous Pony with First Nations youth as a part of the Indigenous Horse-Based Healing process in a way that can help to decolonize existing and future EAS and allow them to be more culturally relevant (17). However, Chalmers and Dell have noted a persistent lack in the incorporation of Indigenous conceptualizations of knowledge in the development and evaluation of EAS (18).

**Table 1:** Terminology, definitions, and example subtypes for equine-assisted services (EAS), equine-assisted learning (EAL), and equine-assisted therapy (EAT).

**Term**	**Working definition**	**Example subtypes**
Equine-Assisted Services (EAS)	Unifying term to refer broadly to services incorporating equines (in this review, specifically horses) in their practices to help human clients^1^	Therapeutic intervenions incorporating horses, learning activities incorporating horses, horsemanship activities
Equine-Assisted Learning (EAL)	Initiatives involving the use of horses with a primary focus on groundwork to promote learning objectives related to academics, personal growth or life/coping skills. Facilitated by instructor certified in EAL methods who may or may not have received additional training in other areas (e.g., mental health promotion).	Equine-assisted learning in education (where goals focus on academic skills, character development and relevant life skills) Equine-assisted learning in personal development (where focus is on strategies to address challenges in one's daily life by developing skills including in communication, effective problem solving, creativity, etc.)
Equine therapy or Equine-Assisted Therapy (EAT)	Therapeutic services which incorporate horses as a metaphor to supplement therapy, or to achieve therapeutic goals. Generally facilitated by a licensed professional trained in a relevant field (e.g., psychology, psychotherapy, social work)	Equine facilitated therapy (EFT), equine facilitated psychotherapy (EFP), equine-assisted psychotherapy (EAP)

*This table has been developed according to the authors' understanding of appropriate definitions in the context of this review, informed by and adapted from Wood et al. (15) and the descriptions and definitions provided by studies included in this review*.

1 *As recommended in a consensus review by Wood et al (15)*.

This review was inspired by the Saakihitiwaac Tipenchikaywin project which targeted at-risk youth who struggled in traditional classroom settings, with a focus on at-risk and First Nations youth in the community of Sioux Lookout, Ontario, Canada. Saakihitiwaac Tipenchikaywin provided proactive and preventative programs and learning therapies, including EAL as one of the activities. The motivation behind this scoping review was to systematically explore the literature to investigate the state of current evidence supporting the use of equine initiatives of this nature with youth with similar experiences, particularly Indigenous youth and at-risk youth more broadly. Given that the Saakihitiwaac Tipenchikaywin project included specifically an EAL activity, the primary EAS of interest in this review was EAL, or activities similar in practice and intention to the initiative in the Saakihitiwaac Tipenchikaywin project. Please refer to section EAL Definition for further details on terminology.

Previous research supports the potential for various types of EAS to address psychological well-being and social and behavioural concerns in young people (19, 20), though methodological concerns with existing research are frequently noted (19–22). Additionally, it has been reported that Indigenous youth have challenges accessing the mental healthcare they require due in part to the monocultural nature of services being offered (23). Snowshoe and Starblanket (17) provide initial evidence that a culturally-responsive Indigenous Equine Therapy program has the potential to have a positive impact on Indigenous youth mental wellness, however the efficacy and appropriateness of animal assisted therapies, including therapies incorporating horses, with Indigenous peoples has not been rigorously studied (24). While health practitioners may wish to provide culturally appropriate care for Indigenous youth, there is often frustration at the lack of empirically-grounded frameworks for providing such care (23). There is therefore a need to further assess the cultural appropriateness of equine therapy initiatives with a range of groups, including Indigenous peoples (24). We were interested in better understanding previous uses of EAL and related programming as initiatives with Indigenous youth populations and learning about which outcomes have been addressed in the literature with an EAL initiative according to our definition.

## Methods

A preliminary search of Google Scholar, PubMed, Cochrane, DARE, Prospero and JBI Database of Systematic Reviews and Implementation suggested there were no previous reviews of the literature specifically on the use of EAS with Indigenous youth as of January 2020. The scoping review followed the framework developed by Arksey et al. (25) and enhanced by Levac et al. (26) as reported by Colquhoun et al. (27) and recommendations by Godin et al. (28) for performing systematic reviews of grey literature. Consultation with content experts was not included due to time restraints. Methodological quality of records was not assessed in this review. A search plan was developed to include both a grey literature search and a review of published peer-reviewed literature in academic databases. First, we developed the research question according to the PICOS framework. The initial *population* of interest was Indigenous youth, however as it was unclear how much literature existed specific to this population, we decided to broaden the population of interest to at-risk youth. This is not to imply that all Indigenous youth are at-risk, but that the broader main category of participants of EAS are at-risk youth, and this was a relevant target group for an evaluation project we were conducting for the Saakihitiwaac Tipenchikaywin project, where most of the participants were at-risk youth and some were also Indigenous. Canada, the United States, Australia and New Zealand were chosen as target countries as the similarities in their colonial histories have made them frequent choices for comparison countries when examining measures of Indigenous well-being (29). As Cooke et al. explain, Canada, the US, Australia, and New Zealand are all highly developed nations with minority Indigenous populations whose health and social outcomes are much poorer than the majority population in their respective country (30). As the initial intention was to focus on Indigenous youth as a target population, these countries were chosen as relevant target countries, and were kept as the focus when the population was changed to at-risk youth. The *intervention* of interest was a limited range of EAS, as defined in the section on EAL Definition. No *comparator* population was used. The *outcomes* of interest were psychological and social outcomes related to mental health and well-being and resilience, as well as cultural and spiritual connection for Indigenous youth. A broad range of *study designs* was considered, including quantitative, qualitative and mixed method original research studies. The final research question can be summarised as: “What types of evidence are currently available to support the use of EAL with at-risk youth?.”

The preliminary review of the literature had revealed that there were many synonymous and related terms to Equine-Assisted Learning (the original search term of interest), including but not limited to: equine therapy, equine-assisted therapy (EAT), equine facilitated learning (EFL), equine facilitated therapy (EFT), equine facilitated mental health (EFMH), equine facilitated psychotherapy (EFP), among others. Given that we were not sure of the scope of existing literature on EAL and related topics, the initial search included as many of these related terms as possible. A working definition of EAL as it will be considered in this review was later developed and is outlined below.

### EAL Definition

As previously mentioned, this scoping review revealed a lack of a standardised definition for EAL or other initiatives involving the use of horses, which has also been noted by other authors (15, 20, 22). Although some attempts have been made to create an operational definition (14), a wide variety of synonymous and related terms were found to be used in the literature. However, we were primarily interested in the use of EAL and similar groundwork-focused programs and their potential for use as an intervention with Indigenous and at-risk youth as this was what was used in the Saakihitiwaac Tipenchikaywin project. Traditionally, EAL is done as group work, with a focus on groundwork and promoting a bond between the horse and the participant that creates an opportunity for experiential learning through structured guided activities involving direct interaction with a horse (31, 32). As mentioned, terms such as EAL and other types of EAS do not have a standard definition and are often used to describe a wide range of interventions. Please see Table 1 for a description of the various equine programs referred to in this review and how they will be considered and referred to in the context of this review. Furthermore, when referring to programs in specific studies, the term associated with the initiative as described by the study authors will be used where relevant. It is also worth noting that where authors of included studies defined their program as “therapy,” we considered this to be the case, even if no specific mention was made of the presence of a mental health professional. For the purposes of this review, a relevant intervention is one that focuses on using groundwork with horses as an initiative with at-risk youth, youth who have experienced trauma, mental illness or family dysfunction, and/or Indigenous youth. Hippotherapy, therapeutic riding and other programs involving only riding as a method of delivering the intervention were excluded. Some programs included both groundwork and some riding activities. The decision of whether to include such programs was made based on the relative amount of groundwork vs. riding activities included and a judgement call based on the focus of the riding activities (e.g., whether these were a final challenge to further concepts established in groundwork or whether the intention was primarily the use of riding as a physical intervention, with groundwork only as a preliminary session). Many programs followed a specific model accredited by a relevant organisation in this area (e.g., EAGALA), however this was not a requirement. Some programs had specific therapeutic goals and were facilitated by mental health professionals, while others were more focused on academic and learning outcomes or on life skills development. Though these initiatives are all quite different in their nature and intentions, we were interested in the range of services addressing these outcomes, and therefore have included a variety of EAS in this review according to the above criteria. As the intention was for interventions to focus on learning and overcoming challenges, initiatives overly focused on physical therapies, or biological or physiological changes were also excluded from the review.

### Database Search Strategy

A university research librarian helped guide the development of both the database and grey literature search strategies. They also provided recommendations of appropriate academic databases to target and search strings to use. The database search was conducted in early February 2020 and included the following databases: Web of Science™ CORE Collection, PubMed, CINAHL, CabDirect^©^, EBSCOHost^©^, and PSYCinfo. The following search terms were combined and searched in the title, abstract, and keyword fields (with the exception of PubMed, where abstract and title were searched, and Web of Science where only title and keyword were searched):

Equine 1^*^: (equine OR horse) AND (therap^*^ OR psychotherap^*^)
Equine 2^*^: (equine OR horse) AND ((facilitated OR assisted) AND (learn^*^ OR therap^*^ OR psychotherap^*^ OR wellness OR intervention^*^ OR “mental health” OR “social work” OR experiential))
**Youth:** “youth^*^” OR “young person^*^” OR “young people” OR adolescent^*^ OR teen^*^ OR child^*^ OR kid^*^
**Indigenous:** Indigenous OR Aborigin^*^ OR autochton^*^ OR “First Nation^*^” OR Inuit^*^ OR Innu OR Eskimo^*^ OR “Alaskan Indian^*^” OR Métis OR Apache OR Cherokee OR Chippewa OR Choctaw OR “Mexican American Indian^*^” OR “American Indian^*^” OR Maori OR Navajo OR Sioux OR “Torres Strait Islander^*^” OR tribe^*^ OR tribal OR (native AND (American^*^ OR Canadian^*^ OR person^*^ OR people OR population^*^ OR man OR woman OR women OR men OR Alaska^*^)) OR “on reserv^*^” OR “off reserv^*^”

^*^These searches were entered as separate searches and then combined to create a search that would include instances of both, e.g., “equine therapy” and “equine-assisted therapy.”

### Grey Literature Search Strategy

The grey literature search was conducted between January and February 2020, following some of the recommendations for conducting a systematic grey literature search by Godin et al. (28), and included a regular Google search, a custom Google search specific to non-governmental organisations and a keyword search of the following grey literature databases: Canadian Research Index, Canadian Electronic Library, New York Academy of Medicine Grey Literature Report, Trip database, US Government Documents Database from Penn Libraries, OAIster^®^, govinfo (United States Government Publishing Office), Canadian Institutional Repositories, Trove, Australian Indigenous Health Infonet, SSRN, Australian Policy Online, SIAhub, and NZ Ministry of Health Grey Matter newsletter. As these resources each had quite different search capacities, it was not possible to use a standardised string of search terms. However, three groups of search terms were used: equine therapy terms (e.g., “equine therapy,” “horse therapy,” “hippotherapy,” etc.), terms referring to Indigenous groups and peoples (e.g., “Indigenous,” “Native American,” “Maori,” “Torres Strait Islander,” etc.) and terms referring to youth and young people (e.g., “youth,” “child,” “teen,” etc.). A variety of search terms and combinations were tested to see which yielded the most relevant results. Relevant results were then exported. If no results were found, search queries became increasingly less targeted, until they had been reduced to the broadest of relevant concepts, e.g., “horse” or “therapy.” In cases where the relevance of the record identified was unclear based on the first glance at the search results, the record was assumed to still have the potential to be relevant and was moved to the next stage of screening. For the general Google search and the customised Google search, the following search queries were alternated: “Indigenous OR youth ‘equine-assisted' location:[X]”; “Indigenous OR youth ‘equine facilitated' location:[X],” where X was either Canada, United States, Australia or New Zealand.

### Screening and Eligibility

The initial search yielded 1,841 search results in the database search, and a further 1,171 in the grey literature search. These numbers represent the total search results identified prior to any screening or removal of duplicates. All identified records from the database search were imported into EndNote X8^©^. The database search was conducted using DistillerSR^©^ review software (Evidence Partners Incorporated, Ottawa, ON, Canada). Grey literature results were first screened independently using Microsoft Excel; those eligible after the first stage of full text review were transferred to DistillerSR^©^.

**Figure 1:**
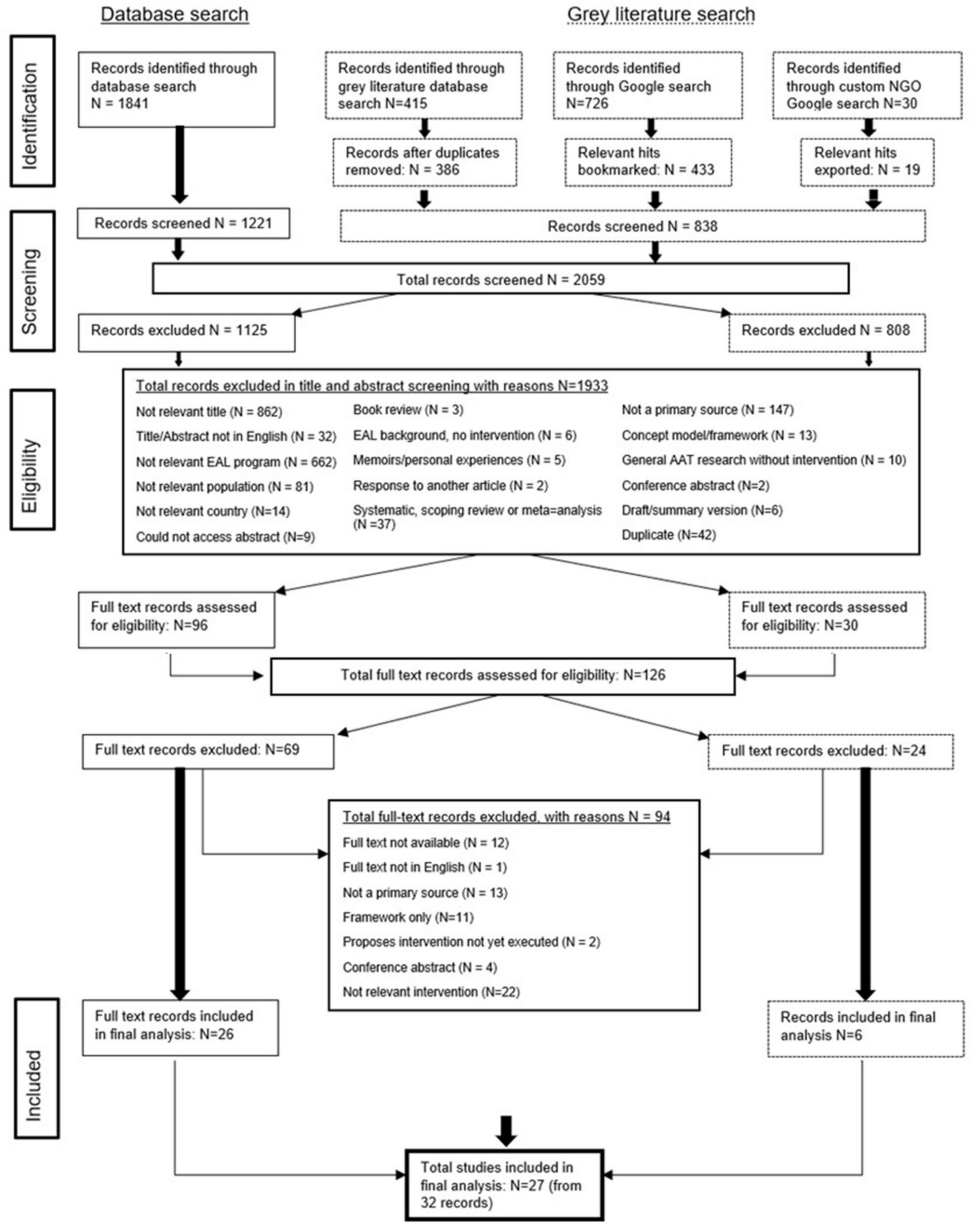
PRISMA flowchart.

Figure 1 shows a flowchart summarising the screening process. Two independent reviewers were involved in the screening process and the determination of eligibility criteria. The search process was not linear; rather the eligibility criteria were re-assessed after each round of screening based on the records remaining. After each round of screening the reviewers discussed how best to adapt the eligibility criteria for the next round. As mentioned, the initial search query and eligibility criteria were quite broad as we were unsure of the scope of existing literature. However, due to the large number of studies identified in the early stages of the review, eligibility criteria were narrowed throughout the process. The two reviewers independently assessed a sample of 10 abstracts on two occasions during the initial screening process and compared findings to ensure consistency in the application of the eligibility criteria. Table 2 outlines the final eligibility criteria. No limitations were placed on year of publication.

**Table 2:** Eligibility criteria.

	**Inclusion criteria**	**Exclusion criteria**
All records	Available in English	Full document not available in English
	Full document available	Duplicate
	Most current version of the document	Document was draft or summary version or was replaced by another document
	Program carried out in Canada, US, New Zealand or Australia	Program not in Canada, US, Australia or New Zealand
	Participants are youth aged 10-18	Participants do not include youth aged 10-18
	Includes relevant EAL intervention^*^	Conceptual EAL framework or model only
		Intervention based only on horse riding
		Book review, opinion piece, memoir, or personal experience only
	Intervention focused primarily on at-risk youth, mental illness/health/wellbeing, trauma, school outcomes, family/cultural connection	Intervention focused primarily on physical disability (e.g., cerebral palsy, spina bifida, etc.), intellectual disabilities (Down's syndrome, autism, ADHD, etc.), hospitalized children, obesity
	Some form of quantitative or qualitative analysis/description of outcomes	Based on biological or physical measures only
		Outcomes assessed based on observations/responses from healthcare practitioners/parents/teachers only
Specific to grey literature	Primary source written document about impact of relevant EAL program on target population	Secondary source (including media reports)
	Author/publisher is a credible organization or individual delivering or evaluating a relevant EAL program^*^ with the target population, (a) participant(s) of a relevant EAL program, OR author/publisher is a stakeholder of the target population who is calling for the need for a relevant EAL program in the target population	Websites promoting programs that are not primary sources or reports
		Does not refer to impact of a specific program

* *Relevant intervention as defined in section EAL Definition*.

### Data Collection and Analysis

The following information was collected for analysis from each record: author(s), publication year, title, country where initiative occurred, target population (including age range), purpose, type of intervention as defined by the study authors (EAL, equine-assisted therapy, equine-assisted psychotherapy, etc.) and a short description of the sessions, description of the intervention facilitators, and model used (if any), research methods used (quantitative, qualitative or mixed methods, and any scales or standardised tests used) and outcomes. Results were collected based on quantitative outcomes reported or summarised based on qualitative analysis. The overall conclusions on the usefulness of the intervention for the given population were noted, as well as any specific findings on the impacts of various cultural and psychosocial measures identified. In accordance with scoping review processes, a formal assessment of the quality of each study was not included.

Several of the included records in the analysis were related to each other, either different records of the same initiatives, or different records of smaller initiatives which were part of the same larger study. However, for the purposes of analysis, the number of studies was used, rather than the number of records (e.g., there could be multiple papers for the same study).

## Results

### Quantitative Results

A total of 27 studies were included in the final analysis informed by 32 records, including 26 records from the database search (24, 31–55) and six records from the grey literature search (56–61). The results of the analysis are summarised in Supplementary Table 1. A total of 12 studies (15 records; 44% of studies and 47% of records) focused on initiatives targeting at-risk youth (35, 36, 40, 42, 43, 51–60), five (19% of studies; 16% of records) targeted youth with mental health disorders or learning disabilities (37–39, 41, 50), six (eight records; 22% of studies and 25% of records) targeted youth survivors of trauma or abuse (44, 45) and four (15% of studies; 13% of records) specifically focused on Indigenous youth (31–34). It is worth noting that many of the studies addressed target populations which intersected analysis categories in this review, for instance several studies targeting at-risk youth also addressed mental illnesses such as depression, and some studies not specifically targeting Indigenous youth still had Indigenous youth enrolled in the program, along with youth of other ethnic backgrounds. The categorisation of studies into groups was done based on what was seen to be the most prominent focus of the program by the reviewers, but these categories should not be seen as mutually exclusive.

Of the 27 studies, 17 (63%) were from the United States [20 records; 63%] (34–45, 47, 48, 50–52, 56, 59, 60), four (15% of studies; 13% of records) were from Canada (31, 32, 49, 57), and six (22%) were from Australia [eight records; 19%] (24, 33, 46, 53–55, 58, 61). No records (0%) from New Zealand met the final eligibility criteria. Many of the initiatives followed program models outlined by various associations or were facilitated by instructors trained according to a certain model. Several of the studies used the same or similar models in their interventions. Nine (33%) of the interventions followed the EAGALA (Equine-Assisted Growth and Learning Association) model (24, 36, 38, 42, 43, 46, 51, 52, 61), four (15%) used either NARHA (North American Riding for the Handicapped Association) (37, 41), or PATH (Professional Association of Therapeutic Horsemanship) (47, 53) (PATH international was formerly known as NARHA), two (7%) used Parelli Natural Horsemanship (54, 55), and two (7%) used methods grounded in Indigenous culture and ways of knowing (31, 32). Other studies used models which were not used by any other studies included in the final analysis, such as HELP (HorsePower Experiential Learning Program) (60), and the Gestalt model (33), among others. A few studies used models specifically tailored to addressing trauma, such as EFT-CT (equine-facilitated therapy for complex trauma) (48) and trauma-focused cognitive behavioural therapy (CBT) (50). Four initiatives (15%) did not clearly identify a model used (34, 56–58).

The included studies used a variety of different methods. Eleven studies (41%) used only quantitative methods (24, 35, 36, 41, 42, 44, 45, 47–50, 56, 60, 61), 10 (37%) used mixed methods^1^ (33, 34, 37, 39, 40, 43, 51, 52, 54, 55, 57, 59), and six (22%) used qualitative methods only (31, 32, 38, 46, 53, 58). Behavioural assessment tools were used by all quantitative studies, which relied solely on these tools for data collection, and all mixed method studies, though one (4%; 10% of mixed method studies) also used surveys for quantitative data collection (54). A range of different combinations of methodologies were used among mixed method studies. Three studies (11%; 30% of mixed method studies) used behavioural assessment tools combined with individual interviews (34, 51, 57), while three others used other methods in addition to these two. One (4%, 10% of mixed method studies) used surveys as an additional quantitative measure (54) with interviews as the qualitative method (55), another also used participant observation (37), and a third used participant diaries in addition to these methods (39). Four studies (15%; 40% of mixed method studies) did not use interviews. Of these, one used only behavioural assessments and participant observation (33), one used behavioural assessment tools, participant observation and focus groups (40, 59), another also used surveys in addition to these methods (43), and the fourth used only participant observation as the qualitative measure (52). Most qualitative studies (*n* = 4, 15%; 67% of qualitative studies) used only individual interviews (38, 46, 53, 58), though two (7%; 33% of qualitative studies) also used participant observation in addition to interviews (31, 32).

There was some repetition of behavioural assessment scales used. Two studies (7%) used both of the same two behavioural assessments, Children's Revised Inventory of Events Scale (CRIES-13) and Human-Animal Bond Scale (HABS) in their methods (44, 45, 47). Notably, these studies also shared one of the same investigators (McCullogh). Additionally, two other studies both used the Behaviour Assessment Scale for Children (BASC) (41, 42), while two more used versions of the Rosenberg Self Esteem Scale (RSES) (34, 54). Depression was a commonly measured outcome among included studies, so many also used behavioural assessment tools specifically designed to measure this outcome. Four studies (15%) included versions of the Children's Depression Inventory (CDI) among the behavioural assessments used (24, 37, 43, 48, 61), while one (4%) used the Major Depression Inventory (MDI) (35, 36) and another used the Beck Depression Inventory (BDI) (24, 61).

### Target Populations

#### At-Risk Youth

There were 12 studies (44%) which specifically identified at-risk youth as the target population (35, 36, 40, 42, 43, 51–60). This was by far the most commonly identified target group among included records, incorporating a range of youth seen to be at-risk of different adverse outcomes. Among the records included in the final analysis, the at-risk category included boys from a residential care community (40, 59), youth receiving inpatient mental healthcare (60), students at-risk of school failure (35, 36, 42, 53), youth at-risk of mental illness (43), youth at-risk due to poverty and/or a history of criminal behaviour and/or abuse (51), youth in foster care (52), youth disengaged from school (54, 55), participants of a youth diversion program (57), youth on probation (56), and youth needing to develop social skills, struggling with school and/or displaying other concerning behaviours, who had not been responsive to other forms of treatment (58). The youth included in these initiatives ranged from ages 6 to 22 years.

All but one study (*n* = 11, 92% of at-risk youth studies, 41% of total) in this category reported positive changes in outcomes overall, although of these, one (8% of at-risk youth studies) did not find statistically significant changes (60) due to reductions in sample size caused by drop out. Idzerda (56) was the only record in this category to report a lack of support for positive outcomes among their study sample of youth on probation. Instead, the initiative was found to have increased maladjustment among participants. Notably, this study found acting out behaviours increased among participants following the equine sessions, which was hypothesised to be related to a possible lack of coping skills among the youth as a result of dealing with traumatic revelations experienced in the sessions (56). However, the findings of the study were ultimately not analysed quantitatively due to the small sample size caused by drop-out (56).

Among studies which found positive outcomes within samples of at-risk youth, common themes included outcomes related to coping and resilience. Several studies found a decrease in reported levels of depression for at least some participants (35, 36, 43). Likewise, improved confidence, self-esteem and self-efficacy was noted among participants in several studies (36, 51–55, 57), as well as increased coping/adaptive, and life skills (42, 43, 53, 57, 60). Some studies also reported improvements in communication and social skills among participants (42, 43, 52, 53, 57). Further, several records in this category expressed the appropriateness for EAS with this population as a way of engaging youth (55, 58), and a particular potential to engage youth who have not previously found success in talk therapy (43).

#### Youth With Mental Health Disorders and/or Learning Disabilities

Five studies (19%) included in the final review focused on initiatives for youth with mental health disorders or learning disabilities. All studies which met final eligibility criteria in this category were American. A wide range of conditions were included in this category, with the general unifying theme of interventions aiming to improve mental health and learning among youth with a special education status or who meet the criteria for a mental illness or disorder. It is worth reiterating that the exclusion criteria limited which types of learning disorders and mental health conditions were included in this review. The age range of participants in this category was 9-18 years.

This category is being addressed separately from both at-risk youth, who may or may not have any official mental health diagnoses, and trauma-focused interventions, although there is likely great overlap between these groups. Additionally, although some of the records in this category included Indigenous youth, and many of the records in the Indigenous youth category addressed mental health conditions, a distinction was made based on the specific focus of the intervention and selection criteria for participants.

Of the five studies included related to mental health and learning disabilities, three of the initiatives (60%; 11% of total) focused on youth with emotional disorders/disturbances (37, 41, 50), with a fourth focused on special education students in general, though students with emotional behaviour disorders were included in this classification, as well as those with other diagnoses such as depression, anxiety, ADHD, autism, FASD, etc. (39). The remaining study focused on youth with concurrent mental health and substance use disorders (38). Two of the initiatives (40%; 7% of total) followed the NARHA model (37, 41), one (20%; 4% of total) followed EAGALA (38), one (20%; 4% of total) used EFP following a trauma-focused CBT model (50), and the final initiative used an equine therapy curriculum created by an AAT provider in Minnesota (39).

The findings of initiatives in this category were mixed. Three studies (60%; 11% of total) found overall positive improvements among participants on at least some measures following the program (38, 41, 50), while the other two (40%; 7% of total) did not find evidence of improvements overall in the analysis of participants' performances due to a variety of reasons (37, 39). Notably, Ewing et al. (37) did not find positive outcomes on most measures assessed, however qualitative observations suggested that participants did benefit from the program and were able to improve in areas of need. The authors suggested several explanations for why these observations may not have been reflected in the overall analysis, including the instability in participants' lives, lower average IQ scores impairing comprehension of tests, and a belief that participants may have been adapted to testing and answered with responses they felt were desirable rather than those which reflected their true feelings (37). Additionally, although Brouillette (39) did not find statistically significant improvements in participant or parent responses, such changes were found among staff assessments. Among the studies which found overall positive outcomes, Stiltner (38) found that the program reduced negative emotions among participants such as feelings of anger, anxiety and depression, and as well that participants found the horses provided a distraction from thoughts of substance use and cravings. Stebbins (41) concluded an overall decrease in externalising behaviours such as hyperactivity and conduct issues, although a significant improvement was not found for internalising behaviours or school functioning. Meanwhile, Roberts and Honzel (50) found EFP to be similarly effective to group therapy in increasing positive moods and reducing negative moods. Qualitative results from Stiltner also suggested participants preferred EAL to other types of therapy as it made treatment feel “more like home” and allowed them to bond and form a trusting relationship with the horses (38).

#### Trauma/Abuse Survivors

A total of six studies (22%) (eight records; 25%) included in the final analysis targeted youth survivors of trauma or abuse, or related issues, such as PTSD. Three studies (50%; 11% of total) identified a target population of youth with a history of trauma (44, 45, 47, 48), while two studies (33%; 7% of total) focused on trauma in family relations, with one study focused on children of parents with problematic substance use (46) while the other focused on children who have experienced intra-family violence (49). The remaining study specifically targeted youth survivors of sexual abuse (24, 61). This group had the broadest age range, with participants ranging from ages 4 to 50, as one study included both youth and adult participants, analysed separately (61). One article in this category compared outcomes between Indigenous and non-Indigenous participants (24), finding the initiative to be equally as effective for both groups.

Two articles in this category (33%; 7% of total) followed the EAGALA model (24, 46, 61), one (17%; 4% of total) followed PATH (47), and one (17%; 4% of total) used EFT-CT (48). The remaining articles (*n* = 2; 33%; 7% of total) were not overly clear in citing a specific model used, referring instead more generally to EAP or EFP as a model, although one further discussed a Diamond Model used by EFMHA (Equine Facilitated Mental Health Association) (44) and the other mentioned similarities to a Gestalt model (49).

All initiatives in this category reported positive outcomes. All seven studies with quantitative methods found significant positive associations between the interventions and outcomes assessed, including a decrease in PTSD symptoms (45, 47), an increase in human-animal bonding (44), decreased depression (24, 48, 61) and anxiety symptoms (24, 48), somatosensory complaints and behaviour dysregulation (48), and an increase in global functioning scores (49). Dunlop and Tsantefski reported increased feelings of safety and security and personal and social development as key themes from their analysis (46). Two studies in this category suggested improvements were greatest among younger participants (ages 4-8 and 8-11, respectively) (49, 61). Schultz et al. further noted a statistically significant greater improvement among youth with a history of physical abuse and neglect compared to those without, and similar although non-significant differences for participants with a history of sexual abuse or with at least one parent with a substance use disorder compared to those without (49). One study found the equine program had similar efficacy to trauma-focused cognitive behavioural therapy used in the control group (47), while another found EFT to have a greater effect on reducing concerning sexualized behaviours in adolescents, compared to standard therapy (24).

#### At-Risk Indigenous Youth

There were four articles included in the final review which focused on Indigenous youth as the primary target population (31–34). Two of these studies were in the Canadian context (31, 32), one was from the United States (34), and one was from Australia (33). Given the differences in the locations of these studies, the sub-populations of Indigenous peoples included also varied. The two Canadian studies focused on the use of EAL with First Nations youth who misuse substances, specifically solvents (32) and volatile substances (31). The American study focused on American Indian Youth and their families (34), while the Australian study focused on providing resources for at-risk Aboriginal youth to help cope with grief, loss or trauma (33). Participants ranged from ages 6 to 25.

Two of the studies used EAL methods specifically adapted to Indigenous culture and needs, with focuses on Indigenous ways of knowing (31, 32). One study followed a Gestalt model, which used a holistic approach to treatment considered culturally appropriate for Indigenous youth (33). For the remaining study, the model used was unclear, although recognising trauma and promoting healing through the incorporation of traditional cultural practices was emphasised (34). Two studies specifically emphasised Indigenous ways of knowing over Western epistemology in the design of their initiatives (31, 32), and all four studies engaged in consultations with and collaborated with the local Indigenous communities at the centre of the initiatives (31–34).

Cultural safety and connection to culture and spirituality were common themes in the outcomes of these studies. In the qualitative analyses the benefits of EAL to facilitating spiritual exchange (32) and improving cultural and spiritual well-being (31) were noted. One study (34) explicitly measured changes in cultural identity using the Native American Enculturation Scale, noting an increase during the program that attenuated at follow-up. Adams noted that the horses facilitated improvements in participant well-being through facilitating the sharing of cultural knowledge, as horses are prominent in the culture of many First Nations in Canada (31). Social functioning and connection were also common themes across these studies. Social adjustment was another frequently mentioned theme, noted in the outcomes of three studies. Social well-being was a key theme identified in qualitative analysis of one study (31), as well as reductions in anti-social behaviour (33) and increases in social adjustment, which were found to have been maintained after 1 year (34). One surprising finding from studies in this category was how learning about horses during the program provided opportunities for participants to learn more about anatomy of the horses, and by extension, humans, and participants' own bodies (31, 32). All four studies found positive outcomes of using EAL with these populations.

### Qualitative Findings

#### The Power of the Horse

Several studies described the power of horses as therapists in such initiatives. The large physical size of horses commands respect from participants (58, 60), which is often something at-risk youth struggle with (60). As they are such large animals, horses cannot be pushed or forced to obey using aggressive methods, which requires participants to seek out alternate strategies of communicating with them (33). Participants had to learn to go beyond using verbal commands with the horses and instead become “in tune with the body language of the horse” (32). Interacting with horses required participants to be assertive without being aggressive, and to have a clear focus and confidence in their actions (39). As Brouillette notes, participants could not simply “fake it” with their horses (39). The large stature of horses may also be intimidating for some participants, forcing them to overcome feelings of fear while engaging with the horses (46, 55, 58, 60). In some cases, the horses were observed to approach participants and initiate the interaction, which was very affirming for participants, suggesting they were worthy of the relationship and connection (33). Many participants reported that the horses made them feel safe and increased their sense of personal security (33, 46, 48, 51). The horses were reported by youth to have a calming presence (32, 46). Horses are often described as being able to mirror participants' feelings and give “honest” feedback (38) as they are highly sensitive animals who respond to the nonverbal behaviour of participants in a way humans often do not (60, 62). Furthermore, youth found the behaviour of horses to be predictable and therefore manageable (46), which may not have been the case with human relationships in their lives.

#### Participant Attitude

As an initiative, EAL appealed to many participants for its different feel and approach compared to both school and traditional therapy. Across the studies included in this review, youth overwhelmingly were reported to have enjoyed the program sessions (31, 33, 34, 46, 55, 57, 59). Interestingly, one study reported that participants felt anger and frustration as a result of the program ending as they missed participating in the sessions (37). In another case, the youth were reported to have asked if the program could be continued after the study finished (34).Youth in an institutional setting reported that the sessions helped them to feel less institutionalised and reminded them of the pets and animals they had at home (38). Participants enjoyed the hands-on nature of the therapy (33, 38) and looked forward to future sessions, which had not been the case in the past (38). Participants were found to enjoy working with the horses (32, 43, 46, 53, 55, 57), and were often observed to be highly engaged during the sessions (43, 58) and willing to try new things (39, 51). In some cases, the participants enjoyed just being with the horses and spending quiet time with them in the moment (31), rather than participating in the EAL activities (32). Notably, this was reported by two of the studies targeting Indigenous youth. Some studies further reported that participants were more engaged at school because of the horse therapy program (33, 55). One study found that the youth reported that they preferred EAL to traditional talk therapy (38), while another suggested it may be a more engaging alternative (43). In addition, participants enjoyed the sessions as an opportunity to be outdoors (31, 33) and found the farm to be a calming environment (57). They also enjoyed the opportunity to learn outside of the school environment (57), with some saying they felt they learned more than they did in school, or the same amount but of a different nature (53). A few studies also reported participants engaged in more healthy habits, including increased physical activity, because of the EAL initiative (31, 55), although this was reported by only a few participants and should be investigated further.

#### Bonding

The bond between participants and the horses was assessed by three studies quantitatively using the Human Animal Bond Scale (44, 45, 47), and noted qualitatively in the results of several others (31, 32, 38, 55, 57, 58). Participants were able to develop a close bond with the horses (31, 32, 38), which was rewarding to them (57). In two related records, a relationship was noted between an increase in measured bond with the horses and a decrease in PTSD symptomology (44, 45), although this was not seen in a third, in part due to the strong bond identified after just one session, which remained consistent throughout (47). Participants often formed a strong bond with one horse in particular (32), and in one study even referred to specific horses from the session as “their” horse (55). Others were reported to refer to the horses generally as their “friends” (32). Youth reported that the horses helped them to feel “welcomed” and “special” (32) and appreciated the non-judgmental connection they felt with the horse (58). Several participants reportedly enjoyed the hands-on nature of the activities with the horses (38). The horses provided an opportunity for safe touch for participants (48) which was especially important for participants who had previous experiences of unhealthy touch and abuse (31, 32). This opportunity for physical touch was also reported to help participants practice compassion and empathy (39) and allowed them to feel heard (58), understood, and important (46). Dunlop and Tsantefski further found youth described the horses as having features similar to a safe attachment figure (46).

#### Exceptional Findings

Although the results of this review suggest largely positive outcomes from EAL programs with at-risk youth, there are some caveats to this. Dunlop and Tsantefski reported on the experiences of one participant who never got over her fear of the horses and was disappointed that she was unable to make many friends in the program (46). When reporting on the initial engagement of youth following a preliminary join-up session with the horse, three observers reported not seeing an engagement with the horse from the participant they had accompanied, although they felt this may have been due in part to the participants only having participated in one session (58). Additionally, Idzerda found a lack of improvement as a result of participation in the equine program, and in fact found participant scores increased in maladjustment and acting-out behaviour compared to the control group (56). This may have been due in part to the very small sample size (four participants total across all conditions) and further exacerbated by the unique difficulties faced by this population of youth on probation. The youth often had difficult home lives and were believed to have limited coping skills to help them deal with emotions revealed through EAL (56). Furthermore, the high rates of reported enjoyment of working with horses by the records included in this review may have been affected to a certain extent by a selection bias, wherein youth who disliked horses did not agree to participate or did not complete the program. Iwachiw notes this possibility in their work, as although all participants reported having enjoyed working with horses, the initiative had targeted youth who like horses during recruitment (43). Therefore, although this review suggests EAL may be an effective and enjoyable intervention for many at-risk youth, individual preferences and situations should be considered. Although these exceptions were not noted among any of the four articles focused on Indigenous youth, they may still be important to consider when working with this population. Depending on their cultural background, it may be possible that some Indigenous youth have not had much previous experience with horses, which could result in some hesitancy towards them. Dell et al. noted for instance that two Inuit participants in their program had never seen a living horse before (32), although no problematic fear of horses was noted. Likewise, the considerations of having sufficient support and coping skills outside of the EAL initiative may also apply more broadly to other at-risk youth, including at-risk Indigenous youth.

## Discussion

The results of this review suggest EAL is an effective treatment program for at-risk youth with a variety of backgrounds and needs, and further reveals limited existing evidence for the use of EAS specifically with Indigenous youth populations. EAL was found to be an effective intervention overall for many at-risk groups of youth to build confidence (46, 52–55, 57), self-esteem (31, 34, 54), social skills (39, 42, 48, 55, 57), communication (31, 32, 43, 48, 52, 53), respect (39, 51), compassion (40, 55), and patience (32, 35, 54, 55), as well as to improve emotional regulation (39, 52, 53), reduce anger (33, 34, 38), and increase self-awareness of feelings (31, 33, 57). Participants were able to learn about building trusting relationships (38, 39, 57–59) and setting and respecting boundaries (31, 48).

These themes have been similarly identified by other reviews of equine interventions. In their systematic review of the impact of equine-assisted interventions of psychological outcomes, Kendall et al. noted increases in self-esteem, self-confidence and empathy, as well as reduced social stress and improved behaviour among the findings of interventions targeting at-risk youth (19). One study included in their review by Bachi et al. did not find statistically significant results, but revealed a trend towards increased positive self-image, trust, life satisfaction and self-control (63). An umbrella review of EAS also noted improved confidence, self-esteem, self-concept and self-efficacy among qualitative outcomes of systematic reviews focused on adults with severe mental illness (studies focused on youth mental illness did not appear to be assessed in the umbrella review) (22). A review of the quantitative and qualitative findings of the use of EFP with at-risk children and adolescents also found improvements in self-esteem, social development, self-control, and reduced maladaptive behaviours among the results of included studies (20).

Due to the generally high engagement levels reported, EAS may be an effective way of engaging youth in therapy who have not been successfully engaged with talk therapy (43). This possibility has also been noted by Kendall et al. (19) and Lentini and Knox (20). Furthermore, the findings of this review support the potential for EAS to be adapted as culturally appropriate therapeutic initiatives with Indigenous youth. Snowshoe et al. discuss the importance of therapeutic interventions for Indigenous individuals to be trauma-informed and spiritually grounded (17), which was reflected in the design and intent of these initiatives (31, 32, 34). All four records included in the study focused specifically on Indigenous youth reported overall positive findings among participants, including facilitating the exchange of spiritual knowledge (32), increased knowledge of traditional teachings (34) and connection to culture and participation in cultural activities (31), increased value of cultural teachings (34), and desire to continue to learn about participants' own traditional culture (34). These outcomes are important given the goal of many Indigenous communities, particularly in Canada, to regain connection to traditional ways of knowing and being (17).

The fact that only four studies focusing on Indigenous youth specifically were identified by this review is somewhat of a limitation and suggests that further evaluation is needed of the effectiveness of EAS with this population. However, despite this many of the findings from other studies included in the review may also be relevant to Indigenous youth. As previously mentioned, some studies for which Indigenous youth were not the primary target population still included participants who identified as Indigenous. The categories of at-risk youth, youth with mental health disorders and trauma/abuse survivors included studies which selected participants based on these life experiences and needs, rather than based on a specific ethnic or cultural background. Though few of the included studies were grounded specifically in Indigenous ways of knowing, many of the initiatives involved broad therapeutic goals such as improving coping skills, resilience, communication, etc. which would likely be relevant to a range of individuals experiencing stressful or difficult life situations. Furthermore, many similar qualitative themes were reported among both Indigenous and non-Indigenous participants across studies.

### Limitations

This review has several limitations. First, only 27 studies, informed by 32 records, were included in the final review and the scope of what was considered a relevant EAL intervention was quite narrow. Furthermore, as previously stated, the quality of the methods of the included studies was not assessed. As the search was conducted via scoping review, the intention was merely to identify existing evidence in the literature pertaining to the research question. Scoping reviews do not seek to critique or evaluate the quality of the methods used in included studies, nor do they include any statistical analysis or meta-analysis of results. Other evidence on the broader psychological and social benefits of equine therapies may not have been included in this review. Gender differences in EAS experiences were also not assessed in this review. Many of the included studies were not gender-balanced and often did not comment on gendered experiences in their findings. Additionally, only texts for which a full English version was available were included. Although this review aimed to include relevant research beyond published, peer-reviewed literature through the inclusion of a grey literature search and a wide range of databases, it is possible that some relevant grey literature may have been missed. There was also often a difficulty accessing the full version of grey literature documents which did not have a commonly accessible digital version, due to restrictions placed on access to libraries and work from home measures implemented due to the COVID-19 pandemic.

### Future Research

The findings around the possibility of increased outbursts following sessions due to a lack of coping skills are compelling, and merit further investigation. As many of the at-risk youth included in these studies were noted to have improved social connection and coping skills following the program, it would suggest these may be areas of need outside of the program setting. It was also reported in some cases that participants were upset by the program ending, or that they requested the program be continued, which although suggests enjoyment of the sessions, may also be indicative of a lack of external support or complete transfer of skills from sessions to be appropriately applied to future challenges. Additionally, several studies experienced small sample sizes due to high rates of participant drop-out, often citing the instability and volatility of participants' personal and home lives. This further suggests participants may lack alternative coping skills which would allow them to effectively process feelings revealed during EAS outside of the program sessions. Overall, this suggests further research is needed into the long-term benefits of EAS, especially outside of a continued program. Future research may also benefit from considering the role of gender in participant experiences and outcomes.

## Conclusion

Although EAS have been growing in popularity in recent years, little is known about the appropriateness of their use with Indigenous youth. This review sought to assess the state of existing evidence supporting the use of equine initiatives for this purpose, through a review of both EAL initiatives with Indigenous youth and with other at-risk youth populations. Overall, EAL appears to be a promising initiative with the potential to be successful with both Indigenous youth and at-risk youth more broadly. Further research is needed into the possible limitations of these initiatives to better predict the individuals and groups where EAL initiatives may not be as appropriate due to a lack of coping skills and external support, as well as a persistent fear of or discomfort around horses.

## Data Availability Statement

The original contributions presented in the study are included in the article/supplementary material, further inquiries can be directed to the corresponding author/s.

## Author Contributions

KS conceived the idea, developed the initial concept for the review, and reviewed and edited the paper. LH performed the initial records search and preliminary screening of articles under the guidance and supervision of KS. LH analysed the findings of the review and wrote the main text, with guidance and ideas from KS. All authors reviewed articles for final inclusion and have read and approved the final version of the article for publication.

## Funding

Funding for this scoping review was provided as part of the evaluation of the Saakihitiwaac Tipenchikaywin project in Sioux Lookout, Ontario, Canada and from a Social Science and Humanities Research Council (SSHRC) Insight Grant (File Number: 435-2017-0926; PI: KS).

## Conflict of Interest

The authors declare that the research was conducted in the absence of any commercial or financial relationships that could be construed as a potential conflict of interest.

## Publisher's Note

All claims expressed in this article are solely those of the authors and do not necessarily represent those of their affiliated organizations, or those of the publisher, the editors and the reviewers. Any product that may be evaluated in this article, or claim that may be made by its manufacturer, is not guaranteed or endorsed by the publisher.

## Footnotes

^1^Fifteen records used only quantitative methods, however, Maujean et al. (55) is not counted with the quantitative studies, despite using only quantitative methods, since it is the same study as Kendall et al. (19) which reported the qualitative findings for this study.
