# Supplementary material for: Use of Equine-Assisted Services to Improve Outcomes Among At-Risk and Indigenous Youth: A Scoping Review
Source: Front Public Health. 2022 Mar 28;10:730644. doi: 10.3389/fpubh.2022.730644 (PMC8996079; doi:10.3389/fpubh.2022.730644)
Supplement: Supplementary file 1 [file Table_1.docx]

| *Author (reference)* | *Year* | *Title* | *Country* | *Target population* | *Age* | *Purpose* | *Type of EAS^[[1]](#footnote-1)^* | *Facilitators of equine activity* | *Model used* | *Description of sessions* | *Methods used* | *Outcomes* |
| --- | --- | --- | --- | --- | --- | --- | --- | --- | --- | --- | --- | --- |
| Adams, B. L.  (38) | 2013 | Using Horses to teach authentic leadership skills to at-risk youth | US | At-risk youth (specifically boys in this study) | 12-15 | “The purpose of this study was to determine the impact of an equine-facilitated authentic leadership development program on at-risk youth.” | EFL^[[2]](#footnote-2)^ | Researcher (Adams, B. L.), horse unit director, and assistant,  older boys, and a professional with a doctoral degree in leadership education | George's model for leadership, EAGALA^[[3]](#footnote-3)^ for activities | “[S]essions covered… different aspect[s] of George’s Authentic Leadership Model (2003):  relationships, self-discipline, purpose, values, and heart.” Taken from EAGALA guide | Mixed methods:  Level of Comfort Questionnaire, demographic questionnaire, authentic leadership questionnaire,  Participant observation,  Focus groups | "A holistic review of the results from both segments of this study demonstrated the program was successful in regards to the objectives of educating youth about authentic leadership." |
| Adams, C., *et al.*  (29) | 2015 | The Helping Horse: How Equine Assisted Learning Contributes to the Wellbeing of First Nations Youth in Treatment for Volatile Substance Misuse | Canada | First Nations youth who misuse volatile substances | 12-18 | “[H]ow do youth experience wellbeing based on their participation in the EAL program?” | EAL^[[4]](#footnote-4)^ | “EAL facilitators”, specific qualifications not specified | Indigenous ways of knowing, culture-based EAL | EAL program focuses on “  relationship, curriculum, formula (experiential learning focus), horse, facilitation and partnerships” | Qualitative,  Participant observation,  Individual interviews,  Diary | The study identified key ways that the EAL program contributed to the well-being of participants through five themes: culture, biological/physical, social, psychological/mental/emotional and spiritual |
| Adams-Pope, B., *et al.*  (57) | 2014 | Horses and At-Risk Youth: An Equine Facilitated Learning Program Focusing on Authentic Leadership Skill Development | US | At-risk boys | 12-15 | “…to teach authentic leadership based on Bill George’s Model (2007). … to find a new way to educate at-risk youth about authentic leadership, using equine facilitated learning.” | EFL | “the horse unit director, the assistant to the director, five  older boys who were assigned to the horse unit, and a professional with a doctoral level degree  in leadership education” | George's authentic leadership model, EAGALA | “[S]essions covered… different aspect[s] of George’s Authentic Leadership Model (2003):  relationships, self-discipline, purpose, values, and heart.” Taken from EFL guides | Mixed methods:  researcher-developed “Level of Comfort” questionnaire, researcher-developed demographics survey, ALQ^[[5]](#footnote-5)^,  Participant observation,  Focus groups | Program was found to be successful in teaching youth about authentic leadership. ALQ showed only a small level of change, but the horse unit director reported positive changes in boys 5 months post program. |
| Bouchard, M.  (55) | 2014 | “I Just Connect With the Horses”: Equine Assisted Learning as a Tool for Developing Social Skills and Resiliency in At-Risk Youth | Canada | At-risk youth | 11-16 | “The purpose of the present multi-strategy study was to describe the experiences reported by at-risk youth in a four-day, activity-based equine assisted learning (EAL) program and evaluate the impact of the program on youths’ social skills and resiliency.” | EAL | Certified riding instructor, two “trained volunteers” and a horse handler | Unclear. Instructor certified by Horse Boy, technique used primarily to improve social and cognitive functioning with ASD^[[6]](#footnote-6)^ | Program lasted four consecutive days and integrated activities from the youth diversion program with equine activities to address social skills and resiliency. Participants developed their own individualized goals for the program | Mixed methods,  Social Skills Improvement System, Resiliency Scales for Children and Adolescents,  Individual interviews | "[P]articipation in an  EAL program may:  1. Contribute to social skills development …  2. Foster protective factors and certain aspects of resiliency …  [I]nterviews with two participants in the EAL program revealed the following … benefits to [participation]:  1. Having fun with horses  2. Feeling an increase in self-awareness, self-regulation, and self-confidence  3. Learning self-monitoring strategies to use at home or school  4. Feeling a sense of mastery and accomplishment  5. Being in a safe and fun environment  6. Developing trust and forming bonds with horses" |
| Brouillette, M. A.  (37) | 2006 | The psychological impact of equine-assisted therapy on special education students | US | Special education students (emotional behaviour disorder, learning disorder, other health impairment as defined by state of Minnesota) | 13-18 | “… this study explored whether a time limited EAT program, had an impact on the psychological strengths and difficulties, prosocial and interpersonal skills of these students.” | EAT^[[7]](#footnote-7)^ | Licensed psychologist | Minnesota Linking Individuals with critters (Animal Assisted Therapy provider) | Focus on addressing “impairment in social skills, classroom  behavior, attention, listening skills, following directions, task completion, organizational  skills, anger control, emotion management, self-esteem, confidence, motivation, learning  interest, empathy, and interpersonal relationship skills” | Mixed methods:  Behaviour rating scales given to program staff (considered qualitative by researcher), SDQ^[[8]](#footnote-8)^ completed by multiple staff, all students and available parents,  Participant observation,  Individual interviews,  Diary | "Findings illustrated that parents and students did not show a significant statistical difference in pretest and posttest scores as a result of participating in the EAT program. However, a significant statistical difference surfaced in staff’s pretest and posttest scores. " |
| Cagle-Holtcamp, K., *et al.*  (49) | 2019 | Does Equine Assisted Learning Create Emotionally Safe Learning Environments for At-Risk Youth? | US | At-risk youth | 6-16 | “Therefore, the objective of this study was to determine if EAL with a focus on educating the youth about the horse creates an emotionally safe environment that promotes learning in at-risk youth” | EAL | EAGALA-certified instructors | EAGALA | Sessions included a practical knowledge section focused on learning basic equine science, practical activities to improve self-awareness and time for processing activities | Mixed methods,  Equine knowledge tests, activity log and self-reporting system to document activities completed each session and goals achieved by participants,  Individual interviews | Participants were all curriculum-compliant and demonstrated improvements in equine knowledge test scores. Participants were found to be confident in their abilities to try new things, expressed positivity about their experiences and an increased sense of personal security. |
| Coffin, J.  (31) | 2019 | The Nguudu Barndimanmanha Project-Improving Social and Emotional Wellbeing in Aboriginal Youth Through Equine Assisted Learning | Australia | Aboriginal youth | 6-25 | “The aim of the current project was to develop a pilot EAL program with at risk Aboriginal youth to determine the effectiveness of EAL in improving social and emotional wellbeing in this demographic” | EAL | Unclear. Appears to include both a mental health professional and equine specialist | Gestalt | Primary objective was “to provide an alternative (resourcing) therapy for Aboriginal  youth in the areas of grief, loss and trauma. … Secondary objectives included increasing school attendance  through rewarding good behavior with a positive therapeutic  experience, and developing a local network of mentorship,  support, and education in the field of equine therapy.” | Mixed methods,  Culturally secure tool based on SDQ, Participant observation using photos | Program found reductions in anti-social behaviour, increases in positive behaviour and body language and anecdotal reports of increased school attendance by teachers, parents and caregivers. Participants reported feeling secure and safe and found the horses to be accepting and non-judgemental |
| Dell, C., *et al.*  (30) | 2011 | A Healing Space: The Experiences of First Nations and Inuit Youth with Equine-Assisted Learning (EAL) | Canada | First Nations and Inuit youth who abuse solvents | 12-17 | “…this exploratory study aims to understand the experience and potential healing impact, if any, of EAL program participation for First Nations and Inuit youth in residential solvent abuse treatment” | EAL | Unclear. Facilitated by EAL facilitators from one equine center and one equine assisted learning center | Aboriginal teaching and ways of knowing | “The …  curriculum is 12 weeks, offered 1 h per week, and is designed to assist youth with  increasing their self-esteem, modifying their behaviour, and healing while having fun”  EAL sessions were accompanied by a journaling session | Qualitative,  Participant observation,  Individual interviews,  Diary | The EAL program gave the youth a space to engage with their culture. It provided an opportunity for spiritual exchange, complementary communication and authentic occurrences between the youth and the horses. |
| Dunlop, K., *et al.*  (44) | 2018 | A space of safety: Children's experience of equine‐assisted group therapy | Australia | Children experiencing problematic parental substance use | 7-13 | “The present study examines child participant perspectives on Horse Club, an EAT intervention delivered by Odyssey House Victoria (OHV), a Melbourne‐based alcohol and other drug (AOD) treatment service” | EAT | An EAGALA trained EAT practitioner and staff from family services organizations trained in social work, psychology, alcohol and other drugs treatment and community welfare | EAGALA | “sessions focused on groundbased  horsemanship activities…  combined with education on equine  behaviour and building participants' somatic awareness and capacity  for mindfulness. In addition …each Horse Club  session began with a group check‐in where children were invited to  discuss how they felt that week, followed by time spent grooming  the horses…  ” | Qualitative,  Individual interviews | Two key themes emerged from the study: feelings of safety and security, and personal and social development. "The present study has found that, for children experiencing problematic parental substance use, horses can assist in providing a safe and secure environment, one filled with enjoyment, in which children are able to make social and personal developmental gains. " |
| Ewing, C. A., *et al.*  (35) | 2007 | Equine-facilitated learning for youths with severe emotional disorders: A quantitative and qualitative study | US | Youth with severe emotional disorders | 10-13 | “This study will explore the use of equines as therapeutic co-facilitators and education enhancers. The purpose of this study is to evaluate the effectiveness of the described method.” | EFL | NARHA^[[9]](#footnote-9)^-certified therapeutic instructors and a volunteer with horsemanship experience | NARHA | Nine-week sessions, two hours in length  twice a week.  Sessions “designed to teach skills such as cooperation, trust, and responsibility” which can be transferred to everyday  interactions. | Mixed methods:  Self-Perception Profile for Children, Empathy Questionnaire, Locus of Control Scale, CDI, Children’s Loneliness Questionnaire,  Participant observation,  Individual interviews | Neither self-esteem, internal locus of control nor interpersonal empathy improved after the intervention and self-reported feelings of depression and of loneliness did not decrease significantly. However qualitative analysis suggested some positive impacts of the program on participants |
| Frederick, K. E.  (34) | 2012 | Understanding the impact of Equine-Assisted Learning on levels of hope in at-risk adolescents | US | At-risk adolescents | 11-17 | “The purpose of this study was to examine the impact of equine-assisted learning on levels of hope, self-efficacy, and depression in at-risk adolescents.” | EAL | “Present at each session were a mental health professional, an equine professional,  the participants, and one or more horses.” | LASSO^[[10]](#footnote-10)^ (EAGALA) | Sessions involved groundwork only. Topics included observation, motivation, attitude, vulnerabilities, and goals, which were taught through various activities with the horses | Quantitative:  the Adolescent Domain-Specific Hope Scale (ADSHS), the New Generalized Self-Efficacy Scale (NGSE), and the Major Depression Inventory (MDI) | “The current study indicates that significant, positive changes in levels of hope, self-efficacy, and depression are seen in at-risk youth who have experienced a five-week intervention of EAL.” |
| Frederick, K., *et al.*  (33) | 2015 | Not Just Horsing Around: The Impact of Equine-Assisted Learning on Levels of Hope and Depression in At-Risk Adolescents | US | At-risk youth | "middle or high school students" | “This study investigates how an intervention which utilizes equine-assisted learning (EAL) impacted levels of hope and depression in participants” | EAL | Unclear | Project LASSO | Sessions involved activities such as observing the horses, trying to catch and halter the horses, and activities focusing on “life’s obstacle”, “vulnerabilities”, and “achieving goals”. Curriculum included setting learning goals | Quantitative,  Adolescent Domain Specific Hope Scale (ADSHS), Major Depression Inventory (MDI) | “The current study shows that at-risk youth were positively impacted by a brief, 5-week EAL experience. Hope levels increased, and depression levels decreased.” |
| Goodkind, J., *et al.*  (32) | 2012 | Feasibility, acceptability, and initial findings from a community-based cultural mental health intervention for American Indian youth and their families | US | American Indian Youth and their families | 7-17 | “The intervention involved a psycho-educational group structure with four main components: (a) recognizing and healing historical trauma through discussion, experiential methods, and traditional cultural practices; (b) reconnection to traditional culture and language through learning from traditional practitioners and elders; (c) parenting/social skill-building; and (d) further healing and building relationships between parents and youth through equine-assisted activities” | Adapted EAP^[[11]](#footnote-11)^ | “The intervention was facilitated by four members of the tribe: two UNM  staff with bachelor’s degrees in psychology, the school-based health center-licensed clinical social worker, and the community behavioral health service preventionist, who was  the only facilitator from the particular Chapter (community) of the tribe”  Monthly horse session components also included a “horse specialist” | Unclear | “[EAP]  occurred monthly (total of six sessions) and involved youth and their parents working  together in groups of six with community horses and a horse specialist to build their relationships  with each other and develop self-awareness and trust” | Mixed methods:  Recent Exposure to Violence Scale, Childhood PTSD^[[12]](#footnote-12)^ Symptom Scale, Native American Enculturation Scale, Harter Self-Perception Profile for Children for children aged 7 to 11, Rosenberg Self-Esteem Scale for ages 12-17, a shortened 12-item version of the Children’s Coping Strategies Checklist, Multidimensional Student’s Life Satisfaction Scale, a shortened version (21 items) of the Social Adjustment Inventory for Children and Adolescents,  Individual interviews | “… for youth who completed at least nine intervention sessions, their traditional cultural identity, self-esteem, positive coping strategies, quality of life, and social adjustment increased. Analyses revealed that increases in child self-esteem, positive coping strategies, and social functioning were maintained at least 1 year post-intervention, while cultural identity, adolescent self-esteem, and quality of life increased and then began to attenuate at the follow-up time points.” |
| Idzerda, A.  (54) | 2009 | Equine Facilitated Mental Health Services and Social Adjustment in Adolescents on Probation | US | Adolescents on probation | 14-17 | “This study was created to determine if participation in six months of weekly involvement in an equine facilitated mental health program would lead to lower scores in an adjustment inventory, indicating greater social adjustment” | Equine facilitated mental health and education services | Unclear | Equine facilitated mental health program at the Esperanza Center | 6-month program of 3 hours of sessions per week. Example activities included spending time with horses (grooming, petting, feeding etc.) discussions of their interactions, drawing their horse, journaling about their experiences and how they faced challenges based on what they learned etc. | Quantitative,  Reynolds Adolescent Adjustment Screening Inventory (RAASI) | "The data from this study does not support the hypothesis that Equine Facilitated  Mental Health and Education programs have a positive effect on adjustment. Unlike other studies that showed significant improvement in behavior, self-esteem, self-efficacy,  or self-awareness, this study instead showed a decline in adjustment in participants who attended the equine facilitated program" |
| Iwachiw, J. S.  (41) | 2018 | A powerful approach or the power of horses: Is equine-assisted psychotherapy an effective technique or the natural effect of horses? | US | Youth at-risk of depression | 11-12 | “Overall, the current study explored the impact of contact with horses on mental health outcomes, specifically targeting youth at-risk for depression.” | EAP | Equine Specialist with EAGALA training and mental health professional with New York State psychology licence | EAGALA | Overarching therapeutic goal was “to improve communication  and problem-solving skills. Each session had a targeted skill and objectives that were associated  with this overarching therapeutic aim… [S]essions for the EAP group targeted nonverbal communication, herd  dynamics and the parallel to human groups and relationships, group functioning, goal-setting,  managing stress and negative pressure, and coping with adversity.” | Mixed methods,  Survey,  CDI^[[13]](#footnote-13)^ 2nd ed Parent and Self Report, demographic survey, treatment acceptability survey,  Participant observation,  Focus groups | “The totality of the results indicates that structured contact with horses may be both beneficial and enjoyable for at-risk youth, and suggest that the use of horses in treatment can result in high levels of treatment engagement, which may then increase learning in that setting for participants. These results suggest that EAP may be an effective alternative technique for participants who do not sufficiently engage with traditional talk therapy approaches. Interactions with horses in this study reduced depressive symptoms for some participants. In sum, either horsemanship or EAP may be effective in reducing symptoms of depression, and an engaging way to develop social skills and coping skills in at-risk youth.” |
| Kemp, K., *et al.*  (24) | 2014 | Equine Facilitated Therapy with Children and Adolescents Who Have Been Sexually Abused: A Program Evaluation Study | Australia | Children and adolescents who have experienced sexual abuse | 8-17 | “The aim of the current study was to evaluate the efficacy of an Equine Facilitated Program used as an adjunct therapy to treat children and youths who have experienced sexual abuse” | EFT^[[14]](#footnote-14)^ | “Counsellors”, exact credentials not specified | EAGALA | Ground-based activities focusing on basic horsemanship skills and activities creating metaphors with the horse to relate to the participant’s life focusing on trust, communication,  boundaries, observation, body language, attitude  and self-perception | Quantitative,  CDI, Child Behavior Checklist (CBCL), Trauma Symptom Checklist (TSCC), BDI^[[15]](#footnote-15)^, Beck Anxiety Inventory (BAI) | "It was hypothesised that participants would show  significant reduction in symptoms of depression, anxiety,  undesirable behaviours and trauma after completing the EFT program. The results supported this hypothesis with  both children and adolescents, regardless of gender or ethnicity, showing a significant improvement in data collected post-EFT compared to scores collected prior to  commencing the program" |
| Kendall, E., *et al.*  (52) | 2015 | Horse Play: A Brief Psychological Intervention for Disengaged Youths | Australia | Disengaged/at-risk youth | 12-22 | “The purpose of this article is to report on the quantitative outcomes of this intervention in terms of levels of self-esteem, self-efficacy, and social behavior relative to a matched control group.” | EFP^[[16]](#footnote-16)^/Natural Horsemanship | Unclear. Sessions involving “mentors” and an ”instructor” and discussions at the end of each week were facilitated by a psychologist | Parelli Natural Horsemanship | Sessions involved a series of 7 structured games focused learning to interact with the horses including consistent non-verbal communication, observation, responsiveness and assertion to build a trusting relationship with the horse. | Quantitative,  Rosenberg Self-Esteem Scale (RSES), The General Self-Efficacy Scale (GSE), Social Behavior Observation Form (SBOF), case manager surveys | "As predicted, participants showed a significant increase in their levels of self-esteem and self-efficacy after participating in the Horse Play program." |
| Maujean, A., *et al.*  (53) | 2013 | Connecting for health: playing with horses as a therapeutic tool | Australia | Disengaged youth | 12-22 | “The purpose of this article is to report on a pilot study of a brief psychological intervention called Horse Play, which was delivered within a program called Connecting4Health.” | EFP/Natural Horsemanship | Mentors, instructor, discussions at the end of each week facilitated by a psychologist | Parelli Natural Horsemanship | 10 weekly sessions which began with a demonstration of natural horsemanship, followed by practice. At the end of each week participants discussed skills they had learned and how these could be applied to their everyday lives. | Qualitative,  Individual interviews | "Based on the interviews conducted with the participants and their case managers, there was strong evidence that the behaviours and skills learned in the program had been translated into the participants’ everyday lives and they demonstrated a positive shift in their overall attitude towards life. The findings of this study lend support to the argument that EFP is a mechanism for providing opportunities to increase the development of life  skills and improve levels of confidence and engagement in young individuals at risk of disengagement and mental illness … Program provided a non-judgemental environment and enabled participants to become competent in tasks." |
| McCullough, L. M.  (42) | 2012 | Effect of equine-facilitated psychotherapy on posttraumatic stress symptoms in youth with history of maltreatment and abuse | US | Traumatized youth (PTSD) | 10-18 | “The purpose of this quantitative study was to investigate whether an EFP intervention leads to a reduction of PTSD-related symptoms in youth who have experienced trauma” | EFP | Therapist, instructor | Diamond model (Equine Facilitated Mental Health Association) | 8 weekly EFP sessions. Sessions included check-ins with therapist and instructor, sometimes including parents, retrieving, grooming and returning the horse, and participation in the activity. In each session, activities focused on creating a metaphor with the horse which was then related to the client’s life. Therapeutic goals were set. | Quantitative,  CRIES-13^[[17]](#footnote-17)^ and HABS^[[18]](#footnote-18)^ | Overall study findings support the assertion that EFP is an effective intervention for maltreated youth experiencing PTSD and that EFP increases human-animal bonding, with 9 out of 11 participants demonstrating increased human-animal bonding and decreased PTSD symptoms at posttest |
| McCullough, L., *et al.*  (43) | 2015 | Equine facilitated psychotherapy: A pilot study of effect on posttraumatic stress symptoms in maltreated youth | US | Maltreated youth | 10-18 | “The purpose of the study was to investigate … if … the levels of symptoms associated with PTSD in youth ages 10–18 change over the course of eight weekly EFP outpatient sessions? … [D]o the levels of the human-animal bond in youth ages 10–18 change …? … [I]s there an association between changes in levels of symptoms associated with PTSD and changes in levels of the human-animal bond …?” | EAP | Somewhat unclear; would appear to include psychotherapist and riding instructor | Object relations/reality therapy | Limited details provided. “Eight weekly EFP sessions, each lasting between 1.5 and 2 hours” | Quantitative,  CRIES-13, HABS | "The results of this investigation suggest that EFP can be effective in lowering PTSD symptomatology in maltreated children and teens. Moreover, the results suggest that the human-animal bond between participant and equine may have contributed to lower PTSD symptomatology scores" |
| Mueller, M., *et al.*  (45) | 2017 | Effects of Equine-Facilitated Psychotherapy on Post-Traumatic Stress Symptoms in Youth | US | Youth who have experienced trauma | 10-18 | “…the objective of this study was to investigate whether EFP is an effective treatment modality for treating post-traumatic stress disorder symptoms in youth having experienced adverse childhood experiences” | EFP | A licensed clinical social worker, a PATH^[[19]](#footnote-19)^-trained therapeutic riding instructor and a trained horse handler | PATH | “The EFP sessions included a variety of activities,  including learning how to lead the horse from field,  grooming, leading the horse in arena and over ground poles,  ground work, and mounted activities.” | Quantitative,  CRIES-13 and HABS | “Findings suggested a significant decrease in PTSD symptoms across the 10-week intervention for both the treatment and control group. However, there was no significant interaction between treatment and time (indicating that the EFP group did not decrease significantly more than the control group).” |
| Naste, T., *et al.*  (46) | 2018 | Equine Facilitated Therapy for Complex Trauma (EFT-CT) | US | Youth with complex trauma histories | 10-12 | “The current study employs empirically-driven clinical case outcome methodology using longitudinal data derived from an agency-designed clinical quality improvement database … three clinical cases of youth who completed EFT-CT will be presented. These cases were derived from a larger EFT-CT pilot (N ~ 20) that is currently in progress” | EFT-CT^[[20]](#footnote-20)^ | Unclear. Appears to include a therapist. | EFT-CT | Focus on safety, attachment and regulation | Quantitative,  Case study data pulled from Client Assessment Tracking System (CATS) which includes UCLA-PTSD Stress Reaction Index (PTSD-RI), Abbreviated Dysregulation Index (ADI), (CDI-2), Child Dissociation Checklist (CDC-3), Adolescent Dissociative Experiences Scale (A-DES), Somatic Awareness Measure (SAM), Child Behavioral Checklist (CBCL), Behavior Rating Inventory of Executive Functioning – Parent (BRIEF-P), Children’s Alexithymia Measure (CAM) | “Across participants in the pilot study preliminary data suggests decreases in anxiety, depression, somatic/sensory complaints and behaviour dysregulation, as well as improved interpersonal skills, communication strategies and overall social functioning” |
| Perkins, B. L.  (50) | 2018 | A Pilot Study Assessing the Effectiveness of Equine-Assisted Learning with Adolescents | US | Adolescents from the foster care system living in group homes | "adolescents" | “…this study proposes the hypothesis that EAL is an effective psychoeducational model. Specifically, this study hypothesizes that the Cowboy Trails program, which involves psychoeducational instruction while working with the horses, would increase life skills of the participants.” | EAL | Mental health counseling graduate student, equine  therapy volunteer, and an EAGALA-certified equine specialist | EAGALA | Sessions included a discussion of the objective for the session (life skill), a task related to this skill to complete with the horses, and time to discuss experiences as a group and draw comparisons to the participants’ lives. Skills included respect, setting boundaries, communication, emotional regulation, problem solving and teamwork. | Mixed methods,  Unnamed 14 question assessment measuring 7 life skills emphasized in the program,  Participant observation | "These results show a relationship between positive behaviors and working with horses among participants in a psychoeducational group." Communication and confidence skills improved throughout the program (although peaked at week 4). Respect also peaked at week 4 but was not found to be significant by the end (week 7). |
| Roberts, H., *et al.*  (48) | 2020 | The Effectiveness of Equine-Facilitated Psychotherapy in Adolescents with Serious Emotional Disturbances | US | Adolescents with serious emotional disturbances | 12-17 | “The current study aimed to examine the immediate effects that equine therapy and traditional group therapy have on adolescents with SED [Severe Emotional Disturbances]” | EFP | Licensed therapist, PATH-certified riding instructor and equine specialists | Trauma-focused cognitive behavioural therapy | Sessions focused on psychoeducation, stress management,  affect expression/modulation, cognitive coping, cognitive processing, behavior  management, and trauma narrative | Quantitative,  Positive and Negative Affect Scale (PANAS) | "The results do not support the hypotheses that EFP would reduce negative affect and increase  positive affect significantly more than would traditional group therapy. However, the results  indicate that participants experienced significant increases in positive affect and decreases in  negative affect in both EFP and group therapy. This result suggests that EFP is similarly effective at increasing positive mood and decreasing negative mood as traditional group therapy" |
| Saggers, B., *et al.*  (51) | 2016 | Horsing around: Using equine facilitated learning to support the development of social-emotional competence of students at risk of school failure | Australia | Students at-risk of school failure | 10-13 | “The aim of the study was to identify how horse care programs may be useful learning tools for students identified as at-risk and to ascertain the value of EFLP^[[21]](#footnote-21)^s in meeting the needs of these students.” | EFL | Facilitators participated in a preliminary accreditation training program by NARHA, supported by teachers and volunteers | PATH | Sessions had weekly focuses including horsemanship, catching and leading horses, communicating with horses, trust building and team building exercises relating to preparing for riding and on horseback, obstacle courses. | Qualitative,  Individual interviews | "Participation was found to further develop resilience in students at-risk. … EFLP horse activities were used to help support and practice many of the essential skills required  to nurture resilience.” Skills included (a) self-regulating  their behavior  (b) making connections and develop friendships with others (c) identifying and developing relaxation and stress-management skills; (d) communicating effectively to  increase their confidence; (e) use of humor and rapport (f) working flexibly and perseverance (g) managing own and others care needs; (h)  planning and successfully carrying out achievable goals, (i) experiencing success |
| Schultz, P. N., *et al.*  (47) | 2007 | Equine‐assisted psychotherapy: a mental health promotion/intervention modality for children who have experienced intra‐family violence | Canada | Children who have experienced intra-family violence | 4-16 | “The purpose of the present pilot study was to test the efficacy of EAP in a cross-sectional group of children referred to a psychotherapist for various childhood behavioural and mental health issues over an 18-month period (June 2003–January 2005).” | EAP | Psychotherapist (licensed independent social worker), equine specialist | Unclear. EAP, possibly similar to Gestalt | Experiential sessions based on metaphors with horses to encourage client insight | Quantitative,  GAF^[[22]](#footnote-22)^ | “Equine-assisted psychotherapy appeared to be effective in improving the GAF scores of children who have been diagnosed with adjustment disorder, mood disorders, PTSD, ADHD [Attention Deficit Hyperactivity Disorder] and disruptive disorders. Young children showed the greatest improvement in GAF scores, and children with a history of intra-family violence and substance abuse tended to show a greater improvement in GAF scores.” |
| Signal, T., *et al.*  (59) | 2013 | Whispering to horses: Childhood sexual abuse, depression and the efficacy of equine facilitated therapy | Australia | Childhood sexual abuse victims | 8-50 | “The current paper had two main aims. Firstly to assess the efficacy of EFT as an adjunct therapy for depressive symptoms presenting in survivors of CSA^[[23]](#footnote-23)^, and secondly, to compare the relative efficacy of this approach across differing age groups, namely children, adolescents and adults.” | EFT | Two “counsellors”, exact credentials not specified | EAGALA | Ground-based activities focusing on basic horsemanship skills and activities creating metaphors with the horse to relate to the participant’s life focusing on trust, communication,  boundaries, observation, body language, attitude  and self-perception | Quantitative,  CDI, BDI | "… EFT proved to result in significant and marked  (i.e., large effect size) decrements in CDI (children) and  BDI (adolescent/adult) scores. ... participants  within the child cohort in the current study evidenced  the greatest average effect size following EFT,  suggesting that this approach may be particularly  effective in reaching younger clients." |
| Stebbins, T.  (39) | 2013 | Effects of an Equine Assisted Activities program on youth with emotional disturbance: A pilot study | US | Special education students identified as "emotionally disturbed" | 9-15 | “The purpose of the present study was to examine the effectiveness of an already established EAA^[[24]](#footnote-24)^ program in improving behavioral, emotional, and academic functioning in youth with ED^[[25]](#footnote-25)^ who were already receiving education and mental health services in a day treatment program” | EAA | NARHA certified instructors | NARHA | Half of each session on horsemanship/groundwork and half focused on riding. “Groundwork riding activities [taught] skills such as compliance with rules, following  directions/directives, verbal and nonverbal communication, active listening, respect  toward horses, instructors, volunteers, self, and each other, and care for horses, among  other skills, while mastering the new skill of horseback riding” | Quantitative:  Behavior Assessment Scale for Children - Second edition (BASC-2) | “Results indicated that, based on teacher ratings, participation in an EAA program had a significant impact on decreasing externalizing behaviors such as hyperactivity, aggression, and conduct problems”  “The present findings indicate participation in an EAA program did not affect  internalizing problems or school functioning.” |
| Stiltner, C.  (36) | 2014 | Equine-assisted psychotherapy in a residential substance abuse treatment program for male adolescents | US | Adolescents with dual diagnoses (mental health and substance use disorder) | 13-17 | “The purpose of this study is to explore the experiences of dually diagnosed adolescent boys who are participating in EAP as part of a residential treatment program.” | EAP | Therapists trained and certified through EAGALA and “horse professionals” | EAGALA | Participants spent one day a week attending EAP group. Activities included leading the horses, getting the horses to maneuver around obstacles, and riding the  horses. Some also participated in individual sessions. | Qualitative,  Individual interviews | Several themes emerged from interviews: 1) the inclusion of EAP made the treatment feel more like home and less like an institution and participants preferred EAP to previous therapy, 2) EAP relieved negative emotions and encouraged positive emotions 3) Participants were able to bond and form a trusting relationship with the horse and looked forward to equine sessions |
| Stock, K. L.  (58) | 2011 | The effect of equine facilitated learning on life skills of youth at- risk | US | At-risk youth 10-15 | 7-16 | “The main goal of the program is to provide hands on experiences for the youth to develop life coping skills that can be generalized into their homes, schools, and communities.” | Equine Facilitated Learning | “Mentors” (Trained in horse safety, horsemanship, mental disorders, positive communication) “handlers” (employees responsible for preparing and leading horses) and “HELP instructor” (certified by NARHA, EAGALA, or had training in a similar background) | HELP^[[26]](#footnote-26)^ | HELP primarily serves youth with behavioural and emotional difficulties. Sessions lasted 1.5 hours a week for 7 weeks. Goal of program is to provide hands on activities to teach coping skills through engagement with horses. | Quantitative:  Children, Youth, and Families at Risk (CYFAR) Evaluation instrument | "Results from this study did not support the hypothesis that participants of the EFL program would demonstrate greater gains in life skills than participants in the control group." Results showed some positive indications in experimental group however they were not significant. Analysis was impacted by sample size and drop out/loss to follow-up |
| Trotter, K. S.  (40) | 2007 | The efficacy of equine assisted group counseling with at-risk children and adolescents | US | "Children and adolescents experiencing behavioural and social skills that place them at-risk for academic success" | 8-14 | “The purpose of this study was to determine the efficacy of equine assisted counseling in decreasing children and adolescents negative maladaptive behaviors, while also increasing children and adolescent's positive adaptive behaviors” | Equine assisted group counselling | Master’s level mental health counsellor, and equine professional experienced in equine assisted counselling | EAGALA | Use of EAGALA activities to incorporate horse as metaphor to stimulate discussions on how group can reach goals | Quantitative,  BASC^[[27]](#footnote-27)^, Animal Assisted Therapy - Psychosocial Form | “Results indicated that participants improved in regards to both internalizing and externalizing behaviours and suggested that the intervention was appropriate for improving adaptive skills.” |
| Waite, C., *et al.*  (56) | 2013 | “It’s different with a horse”: Horses as a tool for engagement in a horse therapy program for marginalised young people | Australia | Marginalized young people | 14-18 | “The program was aimed at young people needing to develop social and communication skills, struggling with school, exhibiting behaviours of concern, and/or those unresponsive to other modes of intervention. ... The goal was to assist these young participants to develop social skills and coping mechanisms by forming an affirming relationship with a horse.” | “Horse therapy” | Youth worker, horse trainer with counselling experience developed through work at a youth agency | Not stated | Participants attempted to establish a “join-up” connection with horse using non-verbal body cues to encourage horse to slow approach and connect with them | Qualitative,  Individual interviews | Participants appeared interested and actively engaged in the join-up and afterwards discussed their experiences enthusiastically. The power differential with the horse in terms of its size, and the non-verbal and non-judgemental relationships participants experienced seemed to appeal to participants. |

Note: This table presents the findings from each of the studies based on what was presented in each of the included papers. There was substantive variation in the level of detail included on each of these various categories, and due to this the table is limited by what was reported by the authors. Some interventions may have included mental health practitioners or established therapeutic goals, however this may not have always been reported.

1. As defined by the study authors [↑](#footnote-ref-1)
2. Equine Facilitated Learning [↑](#footnote-ref-2)
3. Equine Assisted Growth and Learning Association [↑](#footnote-ref-3)
4. Equine Assisted Learning [↑](#footnote-ref-4)
5. Authentic Leadership Questionnaire [↑](#footnote-ref-5)
6. Autism Spectrum Disorder [↑](#footnote-ref-6)
7. Equine Assisted Therapy [↑](#footnote-ref-7)
8. Strengths and Difficulties Questionnaire [↑](#footnote-ref-8)
9. North American Riding for the Handicapped Association [↑](#footnote-ref-9)
10. Leading Adolescents to Successful School Outcomes [↑](#footnote-ref-10)
11. Equine Assisted Psychotherapy [↑](#footnote-ref-11)
12. Post-traumatic stress disorder [↑](#footnote-ref-12)
13. Children’s Depression Inventory [↑](#footnote-ref-13)
14. Equine Facilitated Therapy [↑](#footnote-ref-14)
15. Beck Depression Inventory [↑](#footnote-ref-15)
16. Equine Facilitated Psychotherapy [↑](#footnote-ref-16)
17. Child Revised Impact of Events Scale, 13-item version [↑](#footnote-ref-17)
18. Human Animal Bond Scale [↑](#footnote-ref-18)
19. Professional Association of Therapeutic Horsemanship International [↑](#footnote-ref-19)
20. Equine Facilitated Therapy for Complex Trauma [↑](#footnote-ref-20)
21. Equine Facilitated Learning Program [↑](#footnote-ref-21)
22. Global Assessment of Functioning [↑](#footnote-ref-22)
23. Childhood sexual abuse [↑](#footnote-ref-23)
24. Equine assisted activities [↑](#footnote-ref-24)
25. Emotional Disturbance [↑](#footnote-ref-25)
26. HorsePower Experiential Learning Program [↑](#footnote-ref-26)
27. Behavioural Assessment for Children [↑](#footnote-ref-27)
